# The influence of antenna gain and beamwidth used in OSSEUS in the screening process for osteoporosis

**DOI:** 10.1038/s41598-021-98204-4

**Published:** 2021-09-27

**Authors:** Bruno de Melo Pinheiro, Antônio Luiz Pereira de Siqueira Campos, Dionísio Dias Aires de Carvalho, Agnaldo Souza Cruz, Ricardo Alexsandro de Medeiros Valentim, Nicolas Vinícius Rodrigues Veras, João Paulo Queiroz dos Santos

**Affiliations:** 1 Federal University of Rio Grande do Norte, Campus Universitário Lagoa Nova, Caixa postal 1524, Natal, RN 59078-970 Brazil; 2 Federal Institute of Education Science and Technology, Rua Dr. Nilo Bezerra Ramalho, 1692, Tirol, Natal, RN 59015-300 Brazil

**Keywords:** Engineering, Electrical and electronic engineering, Diseases

## Abstract

Applications on electromagnetic waves in the field of biotelemetry have increased in the latest years, being used to prevent, diagnose, and treatment of several diseases. In this context, biotelemetry allows minimally invasive monitoring of the physiologic, improving comfort and patient care and significantly reducing hospital costs. Aiming to assist the mineral bone density classification, through a radio frequency signal (RF), for a later diagnosis of osteoporosis, Osseus was proposed in 2018. This equipment is a combination of the application of techniques and concepts of several areas such as software, electrical, electronic, computational, and biomedical engineering, developed at a low cost, with easy access to the population, and non-invasive. However, when placed on evaluation, potential improvements were identified to increase the stability of Osseus operation. It is proposed the implementation of improvements in the antennas used by Osseus, aiming its miniaturization, improvement in the reception of the RF signal, and better stability of the equipment's operation. Then, two antennas were built, one of which was used as a project for the second, which is an array. The array showed significant improvements in the radiation parameters relevant to the application, being a candidate to replace the antennas currently in use at Osseus.

## Introduction

Applications of electromagnetic waves in the field of biotelemetry have increased in the latest years. Currently, these waves are used in the prevention, diagnosis, and treatment of a variety of diseases and play an important role in analysis and imaging, thermal therapies, administration of drugs, and sensors^1,2^. In this context, biotelemetry allows minimally invasive monitoring of physiological parameters, improving patient comfort and care and significantly reducing hospital costs^3,4^.

The monitoring of physiological parameters can lead to the diagnosis and, consequently, to the early treatment of diseases, being essential for the implementation of preventive measures. Thus, in the case of osteoporosis, the risk of bone fractures can be reduced^5^.

Annually, millions of bone fractures resulting from osteoporosis happen worldwide. The most common bones with fracture incidence include those of the spine, forearm, proximal humerus, and hips. Hip fractures are those with the highest morbidity and mortality and cause the highest direct costs to the health services, increasing exponentially when they affect the elderly population. It is estimated that in 2050 the number of hip fractures in men and women, within the range of 50 to 64 years in Latin America, will increase by 400%. In Brazil, ten million people, about one in seventeen, suffer from osteoporosis^6^.

To reduce costs with procedures for the treatment of bone fractures caused by osteoporosis, it is essential to implement preventive measures in view of the growing number of elderly people in Brazil and the worsening of the calamity that this disease has caused, hindering early detection and subsequent appropriate treatment^7^. In Brazil, the population has not been given adequate access to early diagnosis of the disease. One of the factors that lead to this is the complexity and high cost of the distribution of equipment throughout the Brazilian territory.

Based on what was exposed, in 2018, Osseus was proposed. An equipment capable of assisting in the classification of bone mineral density, by means of a radio frequency (RF) signal that would lead to a later diagnosis of osteoporosis. This instrument is a combination of the application of techniques and concepts from different areas such as software, electrical, electronic, computational, and biomedical engineering, developed at a low cost, with easy access to the population, and non-invasive^6^.

This procedure is done through the reading of a 2.45 GHz RF signal, which passes through the medial phalanx of a patient's middle finger, and, after the signal is received, it is processed by a system that contains an embedded computational intelligence technique. Analyzing the intensity of the received RF signal, the system can provide a prognosis informing the necessity to refer or not the patient to perform an exam of bone densitometry, or bone density scan (DEXA).

The RF signal of 2.45 GHz was chosen for being an unlicensed frequency band that is high enough to result in small antennas but also because of the background research for Osseus^6^, which also used this frequency band to achieve all of its results in the first place. Thus, for now, we will work only according to this frequency band.

Several patients tested the Osseus right after doing the DEXA exam. They also filled out a list with questions that we considered potentially important to produce a difference in the result of the measures of Osseus (body fat, Body Mass Index, weight, etc.).

Upon being put under evaluation, potential improvements were identified to increase the stability of the Osseus functioning. The main issue was that the intensity of the RF signal varied in the same patient for several measures (and also with patients with similar characteristics and results of DEXA exam), whether due to the positioning of the patient's finger between the antennas, or due to other things like the dimensions of the finger or even its composition (for patients with high or low body fat for example). Another improvement of interest was the manufacturing of equipment with smaller dimensions, in order to increase its portability.

Thus, the physical configuration of the equipment was analyzed, as well as the antennas in use, which in this moment were a pair of Yagi-Uda antennas, fabricated by Kent Electronics Corp. Throughout the research, it was concluded that it was necessary for the pair of antennas used in the transmission/reception to be well-calibrated and to have very similar operating characteristics in terms of signal radiation.

In this scenario, microstrip antennas were a good alternative, as they are known for their miniaturization capacity and simplicity, and low manufacturing cost, despite their narrow bandwidth. However, bandwidth is not a relevant parameter for this application, as no information is being transmitted, but only a RF signal. Other parameters, such as impedance matching and directivity, are essential for the proper functioning of the device.

This article presents state-of-the-art microstrip antennas used in biotelemetry applications. Then we present the main improvements implemented in Osseus, the design of the proposed antennas to be used in the equipment, as well as the main results achieved.

### Latest researches

The use of antennas in biomedical applications is vast, ranging from sensoring to disease diagnosis. In the literature, there are countless researches with proposals for microstrip antennas, in biomedical applications. In this section, we try to identify the main publications in the last 4 years.

Moraes^8^ proposed a miniaturized double spiral antenna for implantable biomedical application at 2.41 GHz frequency band of the Industrial, Scientific, and Medical (ISM) band. Various antenna parameters such as return loss, impedance matching, gain, Voltage Standing Wave Ratio (VSWR), and radiation diagrams were carried. The proposed antenna is fed by the coplanar waveguide method. Only the return loss was measured, with no experimental evidence of other parameters. In addition, an antenna presented low purity of polarization and omnidirectional radiation.

Nachiappan and Azhagarsamy^9^ proposed broadband, low profile, and semi-flexible antenna for biomedical telemetry applications. The antenna was designed in a semi-flexible RT/duroid material. A conventional rectangular patch was modified by adding rectangular slits to reduce the resonance frequency and the ground plane was truncated and modified to increase the bandwidth. This antenna showed a gain close to the commercial ones, about 2.50 dBi at 2.4 GHz and high efficiency, about 93% at 2.4 GHz. Experimental results of the return loss, efficiency, gain, and radiation diagram were presented. Due to the truncation of the ground plane, the antenna showed omnidirectional radiation.

Smida et al.^10^ proposed a triple-band microstrip antenna to operate on the frequencies of 434, 868, and 1400 MHz. The antenna is H-shaped and it was miniaturized, which made impedance matching difficult and significantly reduced its efficiency, falling below 3% in all operating frequencies. In addition, the antenna featured an omnidirectional diagram.

Nikolayev^11^ proposed the design and analysis of miniaturized implantable conformable adhesive antennas for biomedical applications. A polyimide substrate material has been considered to achieve compliance. The proposed radiant element was a rectangular monopolar antenna with three split-ring resonators (SRR) and with coplanar waveguide (CPW) feed. The proposed antenna has an operating frequency of 2.41 GHz, with a bandwidth of 810 MHz and a gain of 2.62 dBi. The authors presented experimental results of the return loss and the radiation diagram.

Ketavath^12^ proposed and designed a circularly polarized implantable broadband antenna operating in the 2.4 GHz ISM band for biomedical applications. Experimental results of the reflection coefficient and the axial ratio were presented. The fractional bandwidth and axial ratio were 5.45% and 5.69%, respectively.

Another circularly polarized antenna was proposed by Yang^13^. The antenna operates in a double band, it is compact and loaded with a metamaterial (MTM), being suitable for biomedical applications. The proposed antenna system operates in the ISM band with central frequencies of 915 MHz (902–928 MHz) and 2450 MHz (2400–2480 MHz). The integration of the antenna with the metamaterial produced an improvement in the gain and behaved as a linear to circular polarization converter, in the two operating frequencies. The gains obtained were − 17 dBi for 915 MHz and − 10 dBi for 2450 MHz. The antenna presented an omnidirectional radiation diagram.

Zada^14^ proposed a clover-shaped split antenna operating in the 2.45 GHz ISM band, for biomedical applications. The proposal aimed at a miniaturized antenna with a wide bandwidth of 180 MHz. The antenna achieved a maximum gain of − 6 dBi. Results measured for the reflection coefficient and for the co-polarization and cross-polarization radiation diagrams were presented. The antenna showed omnidirectional radiation and a very low gain.

All of these researches have antennas with characteristics that do not match the requirements of Osseus, mainly because we need a directive antenna, with a Half Power Beam Width (HPBW) lower than 70° and with a gain greater than 2.5 dBi. In addition, the antenna has dimension restrictions due to the size of the equipment. Thus, in this research we propose an array of two-element microstrip antennas, to reduce the HPBW and provide a satisfactory gain, as well as occupying a reduced space.

## Results

After the designing, the microstrip antenna and the microstrip antenna array, simulations of the main parameters of these antennas were performed in the commercial software ANSYS HFSS, as well as simulations for the old Yagi antenna too. The purpose of these simulations is to compare the performance of the array with the Yagi antennas to make sure the array will be a good substitute for it, making it possible for Osseus to be used. Additionally, the results will also include the microstrip antenna in the comparisons, to verify the effect that the array has on the radiation parameters desired.

### Simulated results

**Figure 1:**
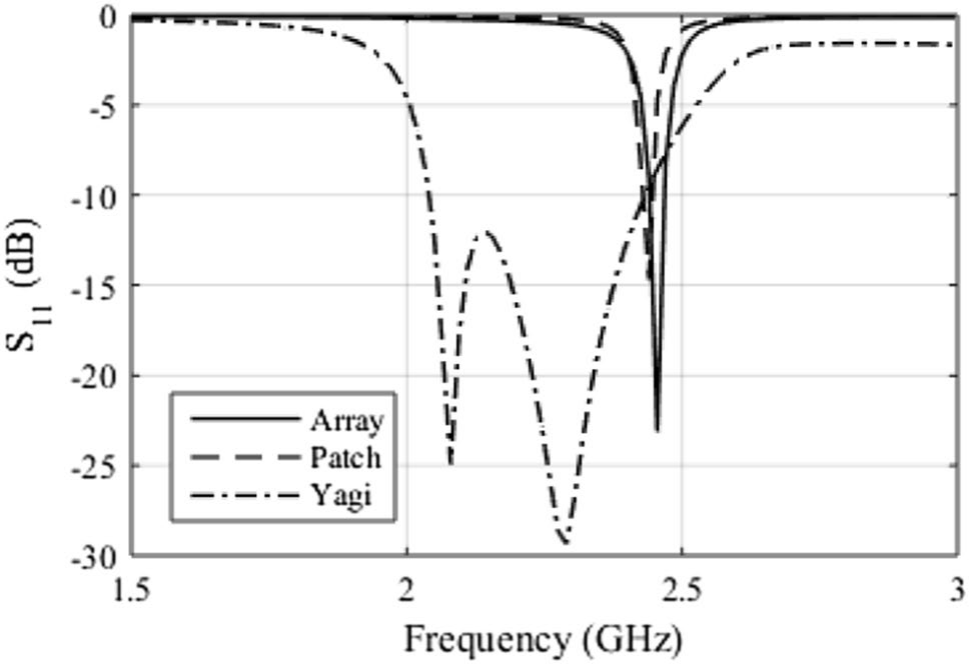
Reflection coefficient for the three antennas analyzed.

The first parameter analyzed is the reflection coefficient. Figure 1 illustrates the simulated results for the three antennas. It can be seen that the Yagi antenna has a resonance frequency equal to 2.29 GHz and a bandwidth of 400 MHz. The patch antenna has a resonance frequency equal to 2.44 GHz and a bandwidth of 15 MHz. The antenna array has a resonance frequency of 2.45 GHz and a bandwidth of 25 MHz. The narrow bandwidth is not relevant to this application. Thus, we observed that the antenna array presents a good match of impedance and resonance frequency equal to that one of WiFi.

**Figure 2:**
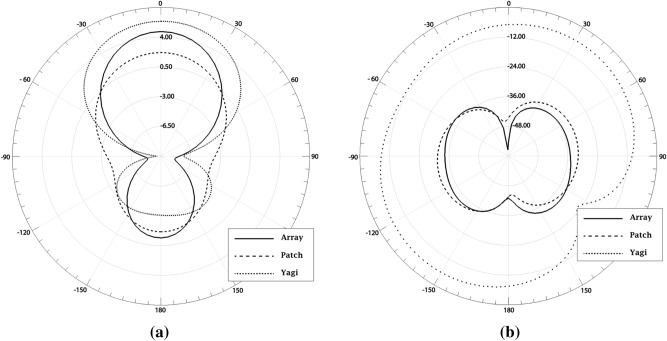
Radiation patterns: (**a**) co-polarization and (**b**) cross-polarization.

Another important parameter that must be analyzed is the radiation diagram. This parameter was simulated for co-polarization and cross-polarization, which are illustrated in Fig. 2. Figure 2a illustrates the simulated radiation diagrams for co-polarization. The Yagi antenna has the highest directivity, around 5.7 dBi, while the patch antenna has the lowest directivity, reaching around 2.5 dBi while the antenna array has 4.7 dBi. However, we can see that the antenna array has a better HPBW of 70°, while the patch antenna has 120° and the Yagi has 95°. Regarding the cross-polarization diagram, illustrated in Fig. 2b, we can notice that the Yagi antenna does not present polarization purity, with a difference of only 16 dB, comparing its co-polarization with the cross-polarization. As for the antenna array, the cross-polarization level reached more than 40 dB below the co-polarization level, which shows a high polarization purity. The patch antenna also features high polarization purity, with a cross-polarization level of more than 35 dB below the co-polarization level.

**Figure 3:**
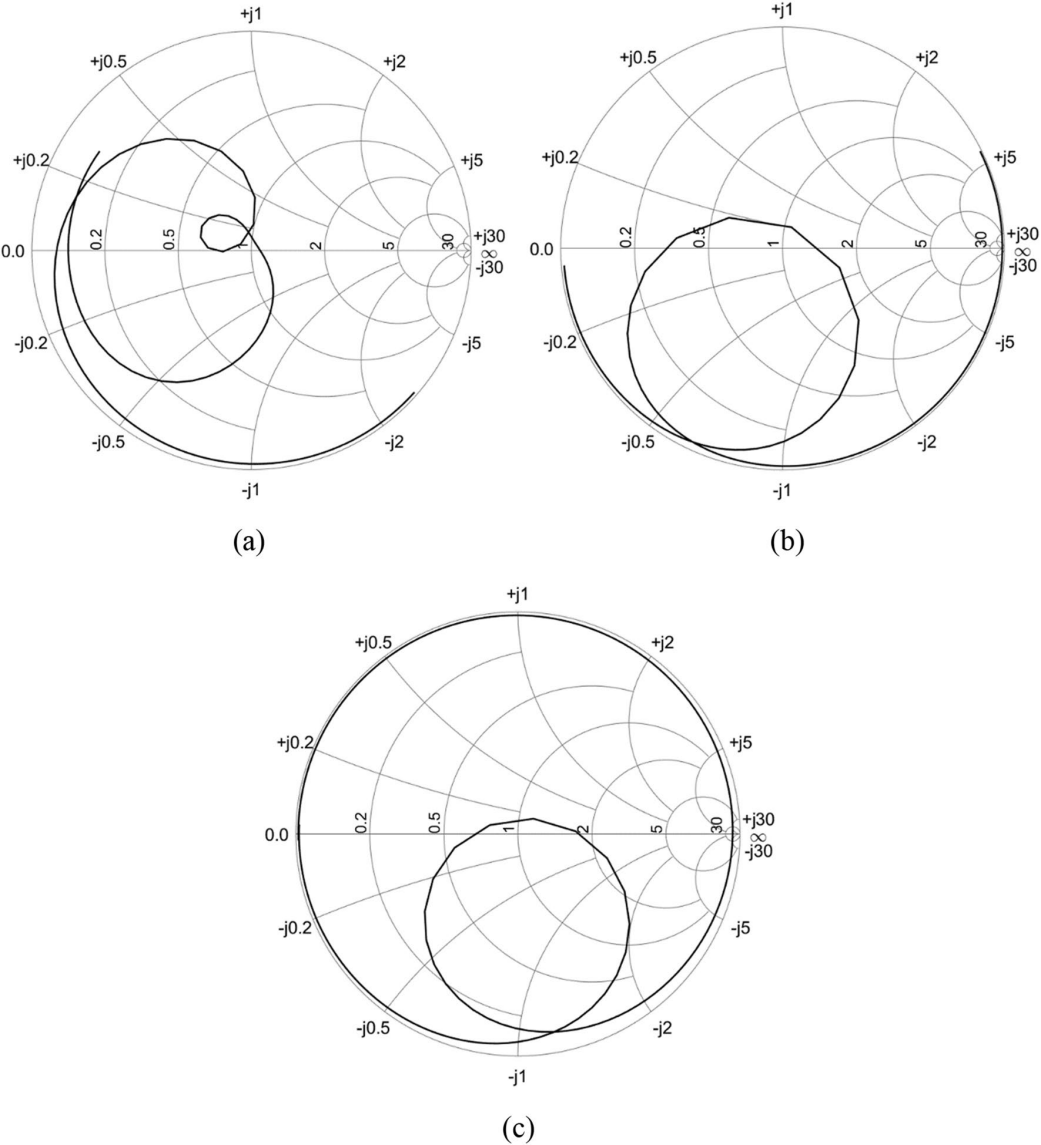
Input impedance at Smith chart: (**a**) Yagi antenna, (**b**) Patch antenna and (**c**) Antenna array.

The next parameter to be analyzed is the input impedance of the antenna, which is shown through a graphic tool, used to display impedance of antennas or transmission lines, called Smith chart. Figure 3 illustrates the Smith chart of the three antennas for a range of 1 GHz around the target frequency, which is 2.45 GHz. For the Yagi antenna, the Smith chart is illustrated in Fig. 3a. It can be seen that the resonance occurs at 2.29 GHz and the normalized impedance was 1.065 + j 0.025 Ω. For the patch antenna, the resonance frequency occurs at approximately 2.45 GHz, but it can be seen in Fig. 3b that the normalized impedance of the antenna does not cross the unit circle at this frequency, showing the need for improvement in the matching of antenna impedance. Finally, considering the antenna array, the resonance frequency occurs at approximately 2.45 GHz. The normalized impedance of the array crosses the unit circle, showing a good design of the feed network, with an input impedance of 49.5 − j7 Ω (Fig. 3c).

Table 1 shows the comparison between the Yagi antenna, patch antenna, and antenna array, according to the parameters shown.

**Table 1:** Parameters of the antennas analyzed for their resonance frequency.

Antenna	Parameter	Value
Yagi-Uda	Directivity	5.7 dBi
	Bandwidth	390 MHz
	Half power beam width (HPBW)	95°
	Front-to-back ratio	5.41 dB
Patch antenna	Directivity	2.52 dBi
	Bandwidth	16.3 MHz
	Half power beam width (HPBW)	120°
	Front-to-back ratio	3.3 dB
Antenna array	Directivity	4.7 dBi
	Bandwidth	25.4 MHz
	Half power beam width (HPBW)	70°
	Front-to-back ratio	5.1 dBi

Based on the data listed in Table 1, we can see that the antenna array has a lower performance than the antenna currently in use in Osseus, as the patch antenna too. However, the proposed array has smaller dimensions than the Yagi antenna used. While the array has dimensions of 3.5 cm × 7.0 cm, the Yagi antenna has dimensions of 6.6 cm × 7.0 cm. Both have equivalent performance in terms of directivity and front-to-back ratio. The antenna array has a lower bandwidth, but that is not relevant for the application. In addition, the array has a lower HPBW, which can promote better results and an improvement in equipment performance, since stability is an issue. Finally, the way in which the coaxial cable is connected to the antenna array eliminates the instabilities found in the Yagi, which can prove to be a significant improvement. Another fact to be noted is that the Yagi antenna is positioned along the longest axis of the Osseus equipment, while the antenna array will be placed perpendicular to that axis. In this way, the antennas will operate in the distant field region. This will prevent variations in the reading of the signal intensity from occurring.

**Figure 4:**
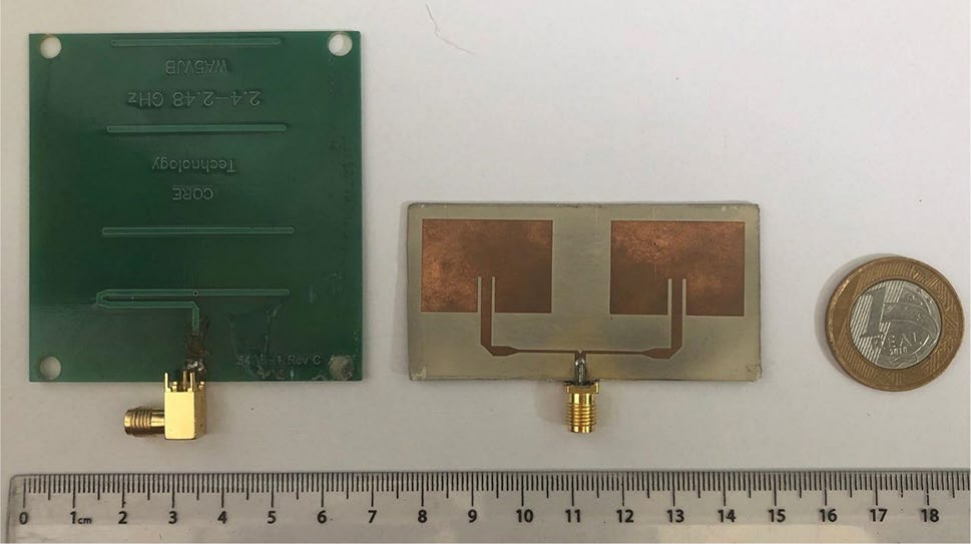
Yagi antenna currently in use compared in scale to the antenna array.

From the decision of the antenna that would be used in the equipment, some prototypes were manufactured for experimental validation and to analyze the improvements in the equipment. Figure 4 illustrates the Yagi antenna currently in use compared in scale to the built antenna array.

### Measured results

The measurement setup was set up with various equipment. The Agilent two-port network vector analyzer, model E5071C, with a measurement range from 300 kHz to 14 GHz was used. Cables appropriate for this frequency range were used. In addition, for gain measurement, we used standard horn antennas model SAS-571 (double ridge guide horn antennas). We tried to measure all possible parameters, with the available setup. The parameters measured were: the modulus of the reflection coefficient (|*S*_11_|), gain of the array, and the Smith chart.

**Figure 5:**
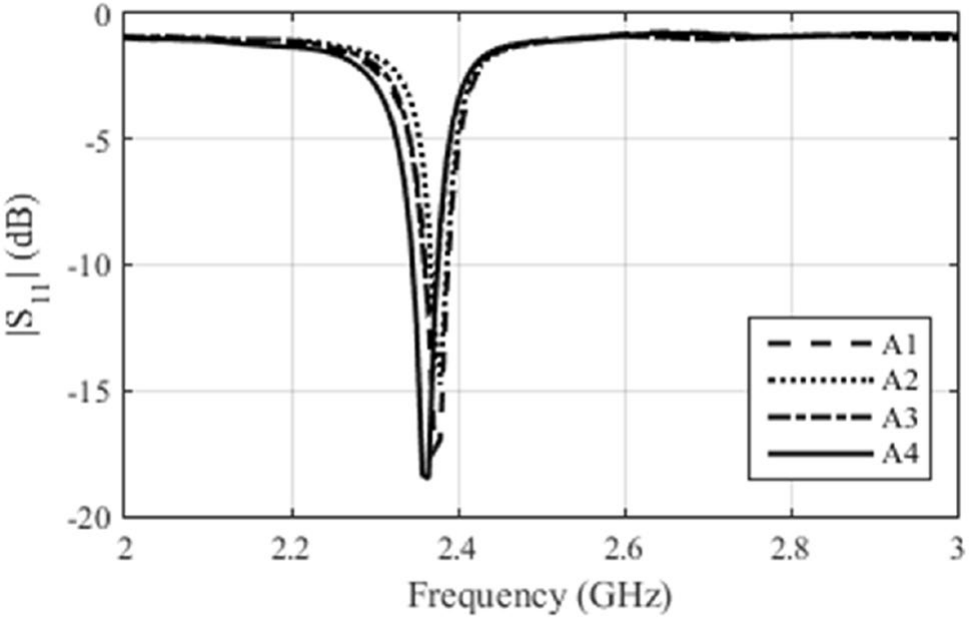
*S*_11_ measure for the four constructed antenna arrays.

**Figure 6:**
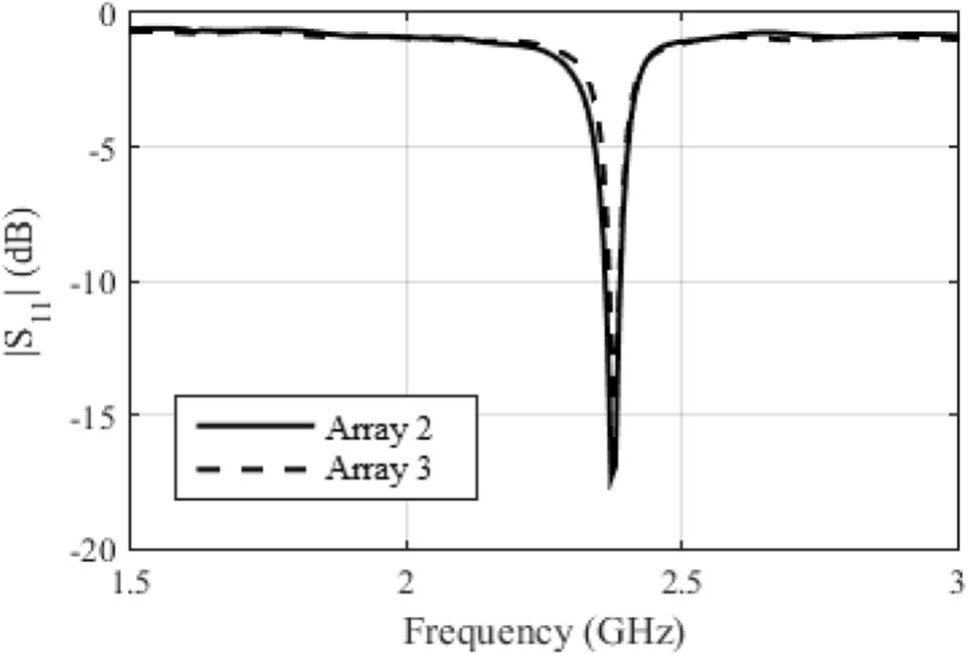
*S*_11_ for the antenna arrays 2 and 3 constructed.

The first parameter measured was *S*_11_, which allows us to analyze the resonance frequency of the antenna and its bandwidth. We measured this parameter for four manufactured arrays. Figure 5 illustrates this parameter. We can see that the antennas responded in a similar way. The antenna arrays that presented the best impedance matching were installed in Osseus. Figure 6 illustrates the frequency response of *S*_11_ only for these two arrays. We can see that the results are very similar.

**Figure 7:**
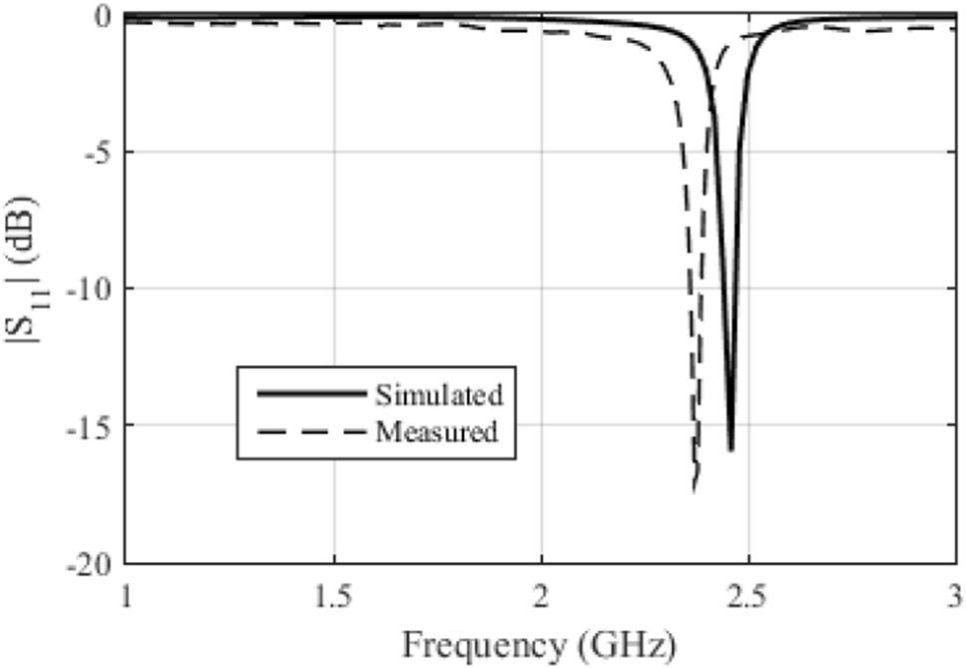
Comparison between the simulated and measured *S*_11_ for antenna array 2.

In Fig. 7, we compared the simulated S11 with the measured response for antenna array 2. We can see that there was a small difference between the results. This is perhaps due to the manufacturing process, which was not done with a milling machine. This difference is not a problem, as *Osseus* has a voltage-controlled oscillator, whose oscillation frequency can be regulated between 2.25 and 2.65 GHz.

**Figure 8:**
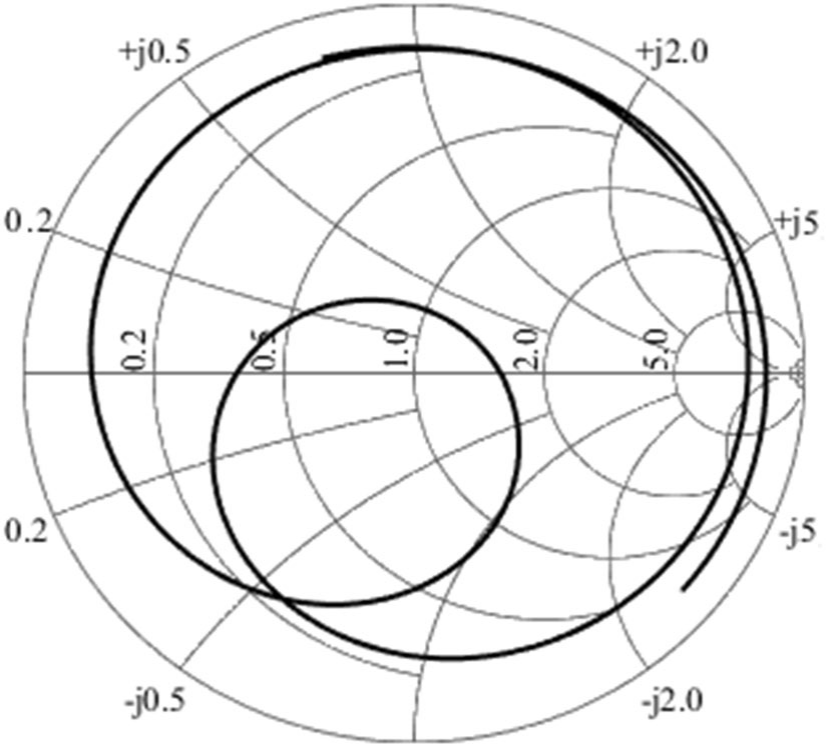
Input impedance at Smith chart for constructed antenna array 2.

Figure 8 illustrates the measured input impedance at Smith chart. We can see a reactance greater than that obtained in the simulation, which was 7 Ω, while the measured value was 12 Ω. This difference represented in the reactance caused a reduction in the resonance frequency.

**Figure 9:**
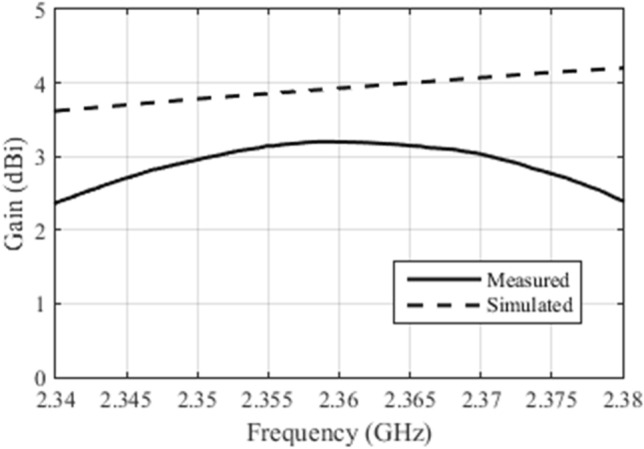
Comparison between the simulated and measured gain for the constructed antenna array 2.

Figure 9 shows the comparison between the simulated and measured gains for antenna array 2, near the value of maximum gain.

## Discussion

**Figure 10:**
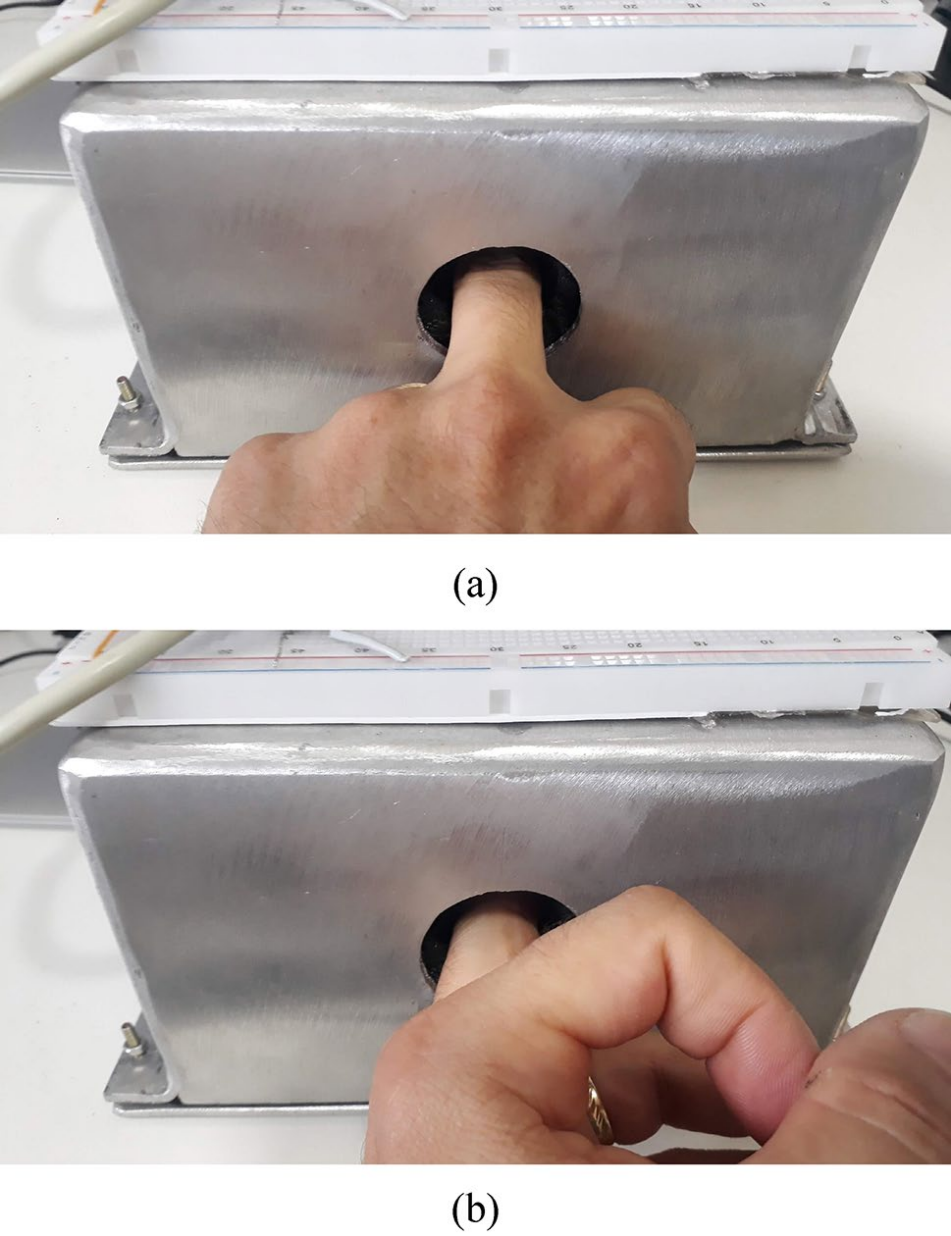
Insertion of middle finger in positions: (**a**) correct and (**b**) rotated.

One of the main improvements that needed to be made in Osseus was to reduce possible variations in the reading of the signal intensity. It is important that the signal-to-noise ratio (SNR) received remains constant, or that it undergoes minimal changes. This will allow computational intelligence tools to achieve a more accurate classification when doing the screening. It is observed that the previous version of the Osseus, which used the Yagi-Uda antennas, presented considerable SNR variations, depending on the position of the finger inserted in the equipment. To perform the measurements, two positions of the finger were considered, as shown in Fig. 10. The first measurement is made with the finger inserted in the position named “correct” (Fig. 10a) and the second measurement is made with the finger inserted in the position named “rotated” (Fig. 10b). This will cause the RF signal to reach a greater or lesser finger surface. The desired result is that the SNR does not vary, even with the finger inserted in a rotated manner, allowing a more stable reading. One of the main reasons for these improvements was the reduction of the HPBW which resulted in a narrower beam that focused the radiation in the finger. This characteristic has a proportionality relation with the number of radiating elements in a parallel array because the sum of the waves produced by each element has constructive collisions in the direction of propagation and tends to have more destructive collisions in the other directions (or side lobes if we look at a radiation pattern).

The measurement setup, taking Fig. 10 as an example, is composed of an aluminum box, which is internally revested with a microwave absorber material (Eccosorb®) fabricated by Techni3, that will mainly protect the measurements from the noise caused by reflecting waves inside the box, a pair of antennas placed on both sides of the box, pointing to each other and the electronic circuit that will inject the signal in the transmitting antenna and compute it after received by the receiving antenna.

**Figure 11:**
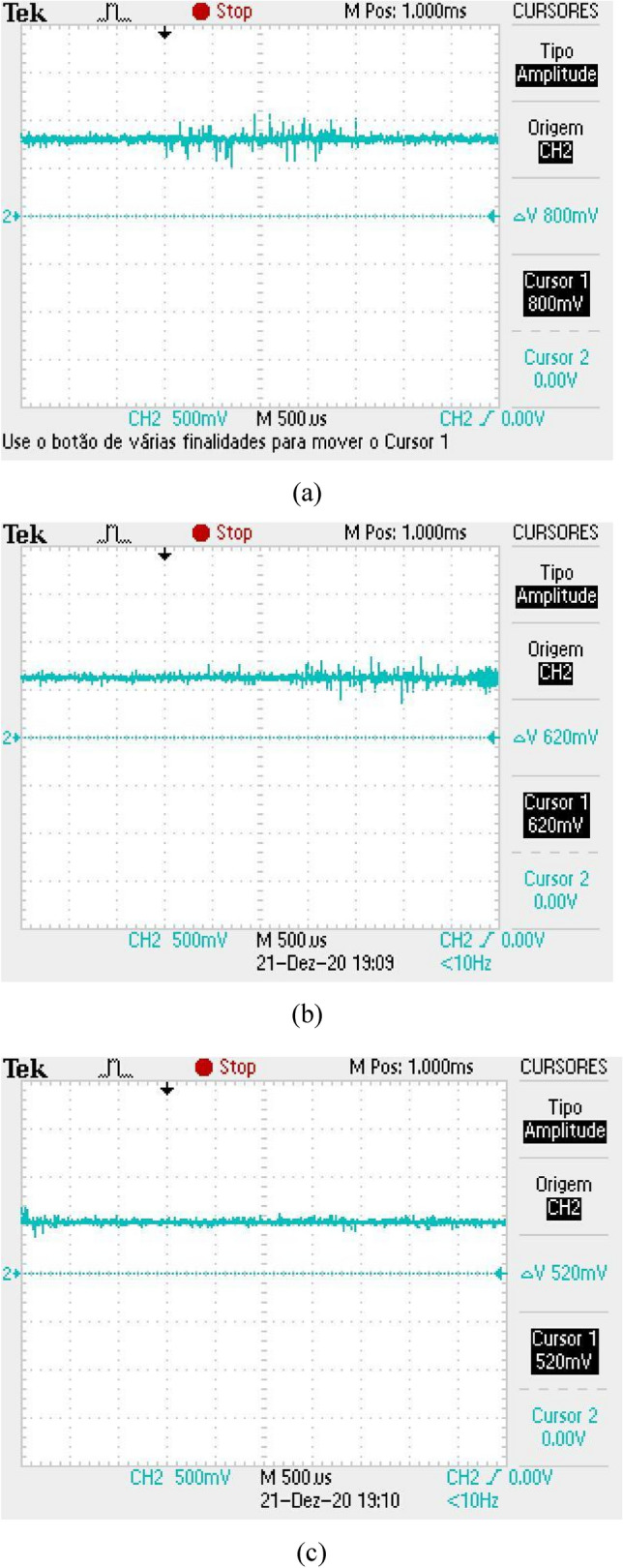
Reading of the DC level of the received signal for the first version of the *Osseus*: (**a**) without finger, (**b**) with finger in the correct decision and (**c**) with the finger in the rotated position.

**Figure 12:**
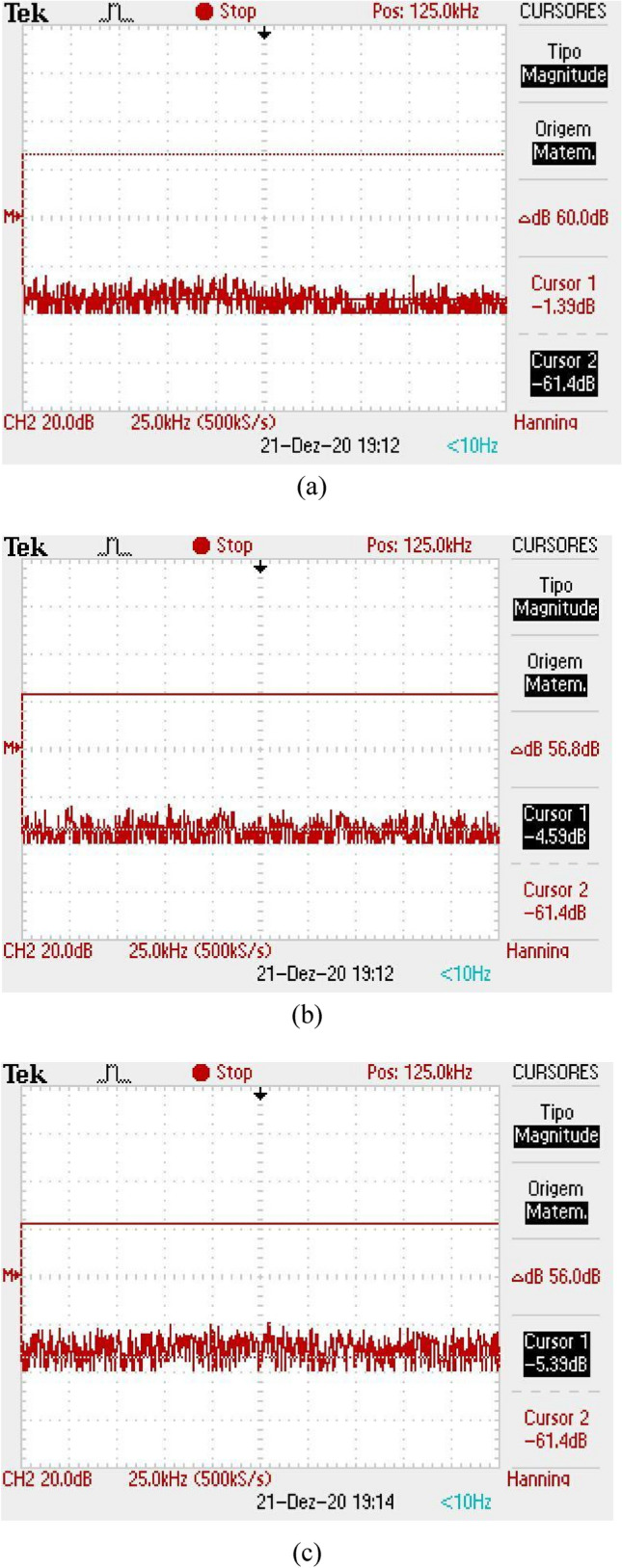
Reading of the noise level in frequency domain for the first version of the *Osseus*: (**a**) without finger, (**b**) with finger in the correct decision and (**c**) with the finger in the rotated position.

The received signal is obtained in a form of a level of direct current (DC) with a certain level of background noise coupled to it. The DC signal has a maximum level when there is no obstacle between the antennas and this value gets lower when the finger is inserted into the equipment. Also, a shield made with absorbers was inserted inside the box, so that the noise level remains constant, regardless of the reading, with or without the finger. Figure 11a illustrates the DC level received for a direct reading (without the finger insertion), for the case of the first version of Osseus, with the Yagi-Uda antennas. In Fig. 11b,c we can see the readings with the finger insertion in the correct and rotated position, respectively. It can be seen that there is a considerable level of noise, in addition to noticing a variation in the level of the received signal, for the different positions of the finger. Without the finger, the received signal is about 800 mV. For the reading with the finger in the correct position, the received signal is 620 mV, while for the reading with the finger in the rotated position, the received signal is 520 mV. In the frequency domain, we can verify whether the noise varies or not. Thus, the same measurements were made and the results can be seen in Fig. 12. The noise level for the three situations considered above was − 61.4 dB, with no variation for the situations with or without the finger.

**Figure 13:**
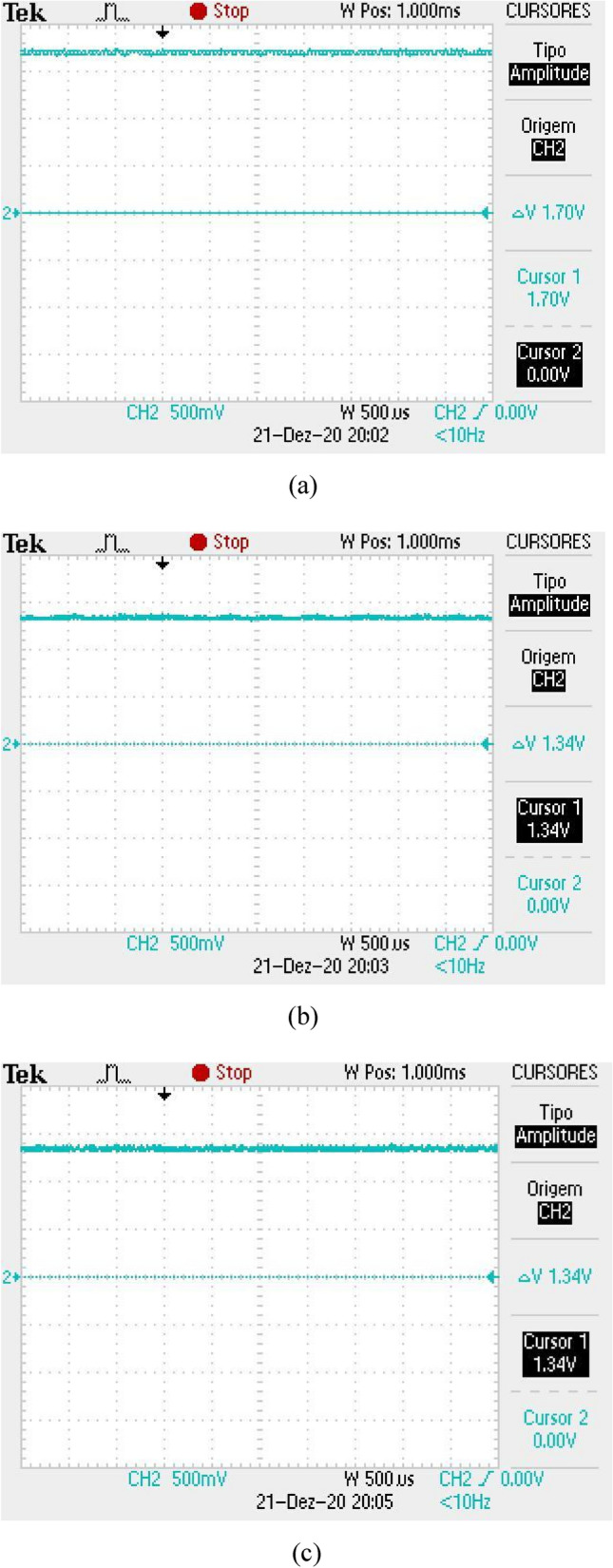
Reading of the DC level of the received signal for the second version of the *Osseus*: (**a**) without finger, (**b**) with finger in the correct decision and (**c**) with the finger in the rotated position.

**Figure 14:**
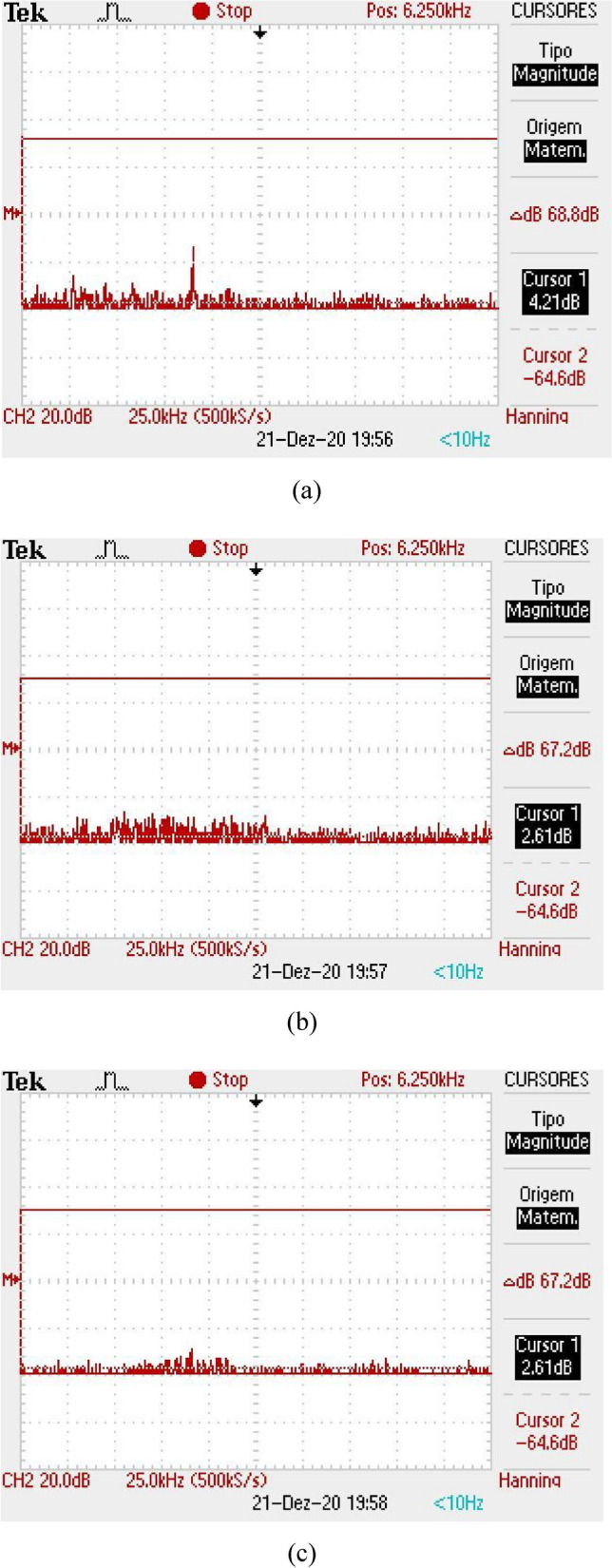
Reading of the noise level in frequency domain for the second version of the *Osseus*: (**a**) without finger, (**b**) with finger in the correct decision and (**c**) with the finger in the rotated position.

The same measurements were made for the second version of Osseus, with the antenna array. Figure 13a illustrates the DC level received for reading without the finger and in Fig. 13b,c we have the readings with the finger inserted in the correct and rotated position, respectively. It can be seen that the noise level has decreased considerably, in addition to the fact that we no longer have a variation in the level of the received signal, for the different positions of the finger. The received signal intensity was also higher. Without the finger, the received signal was 1.70 V. For the reading with the finger in the correct and rotated positions, the received signal was 1.34 V. In the frequency domain, we can verify that the noise did not varied. Thus, the same measures were made and the results can be seen in Fig. 14. The noise level for the three situations considered above was − 64.6 dB, a reduction of 3.2 dB, compared to the first version of Osseus.

Based on the measurements made, it was possible to obtain the signal-to-noise ratio for all situations considered, for both versions of the equipment. Table 2 illustrates the SNR values obtained for each situation considered. We can observe an increase of more than 8 dB in the SNR, in all situations, which represents a very significant improvement.

**Table 2:** Measurements of SNR for the two versions of *Osseus*.

Version of *Osseus*	Situation	SNR measured ( dB)
First	Without the finger	26.03
	With finger in correct position	24.66
	With finger in rotated position	23.32
Second	Without the finger	34.83
	With finger in correct position	33.22
	With finger in rotated position	33.22

## Conclusions

The antennas of the first and second version of Osseus were characterized to find out if they were the most appropriate for the equipment. The main parameters of the antenna were simulated in the HFSS software. It was observed that there is no uniformity of these parameters within the entire operating range. The antennas showed a satisfactory gain in the resonance frequency, but this gain suffered a great variation throughout the operating range. In addition, the HPBW of the antenna proved to be high.

A new antenna configuration was proposed, aiming to improve some performance parameters, such as directivity and HPBW. For this, a classic design was proposed to be used in Osseus. The results of a simple rectangular patch antenna with inset fed were shown. In addition, an antenna array of the same geometry was designed with two microstrip patch antennas. It was noticed that the array has a performance similar to the Yagi-Uda in the first version of OSSEUS. However, the array has a lower HPBW and smaller dimensions when compared to the Yagi antenna. The kind of array designed reduces the problem of connecting the coaxial cable to the antenna. The main parameters of the antenna and array were simulated in the HFSS software. Four prototypes of the proposed antenna array were built, aiming at tests on the Osseus equipment, as well as the measurement of the antenna parameters, such as *S*_11_, gain, and input impedance.

The measurements showed that the prototypes of all antenna arrays constructed showed similar performances, regarding the measured parameters. We selected the two best arrays to be installed in *Osseus.*

The insertion of new antennas brings significant improvements for the Osseus, not only reducing the size of the Osseus box, but increasing the intensity of the received signal and reducing the intensity in the noise level. This is because of the small beam width combined with better directivity. The signal-to-noise (SNR) increased by more than 8 dB. These improvements will allow for more accurate classification of the equipment, once the Osseus is now more stable and reading became independent from the position of the patient's finger, which is something desirable, so as not to interfere with the diagnosis. With the Yagi-Uda antennas, this was not possible. The reading was dependent on the patient’s finger position.

## Methods

The first part of the research aimed to identify possible improvements in *Osseus*. Thus, characterizing the antennas used previously was an important action to start up this task. The antenna used a Yagi type, which is a directive antenna, with a gain of approximately 4 dBi. The input impedance of the antenna is 50 Ω, which allows easy matching for most of the feed networks.

**Figure 15:**
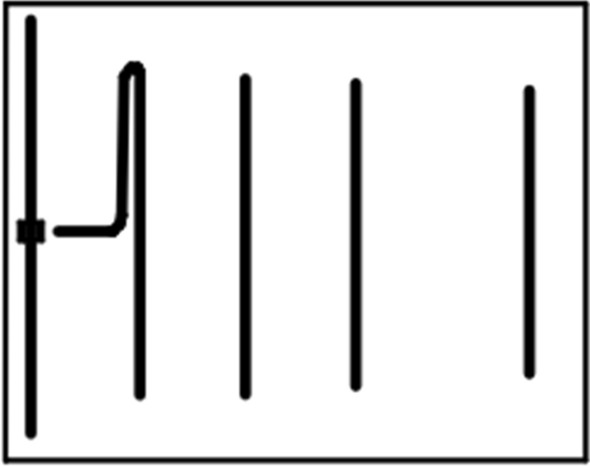
Yagi-Uda antenna analyzed.

The Yagi antenna is illustrated in Fig. 15. It has a 50 Ω single-ended feed input and this transmission line is converted into a two-wire feed through the conductive path.

**Figure 16:**
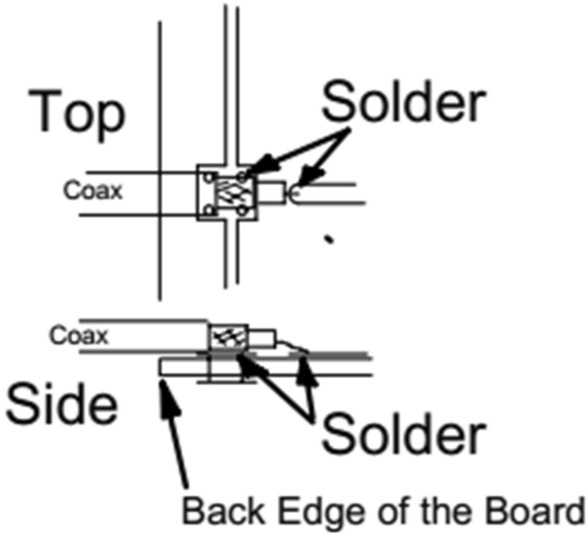
Connection between the coaxial cable and the Yagi-Uda antenna analyzed.

One of the main problems of this antenna is the way that the coaxial cable is connected to it. Figure 16 illustrates this form of connection. It can be seen that a minimum error in welding can lead to an impedance mismatch changing the way it operates. The ground mesh must be welded on the reflector part and the central conductor of the coaxial cable must be welded on the dipole.

Another problem refers to the region in which the electromagnetic field of this application is located. This type of antenna must operate in the distant field region, that is, the two antennas must be separated by a distance of 2R, where R is calculated as:

1 $$R\succ \frac{2{D}^{2}}{\lambda }$$

In which *D* is the largest linear dimension of the antenna and *λ* is the wavelength of the antenna's resonance frequency.

**Figure 17:**
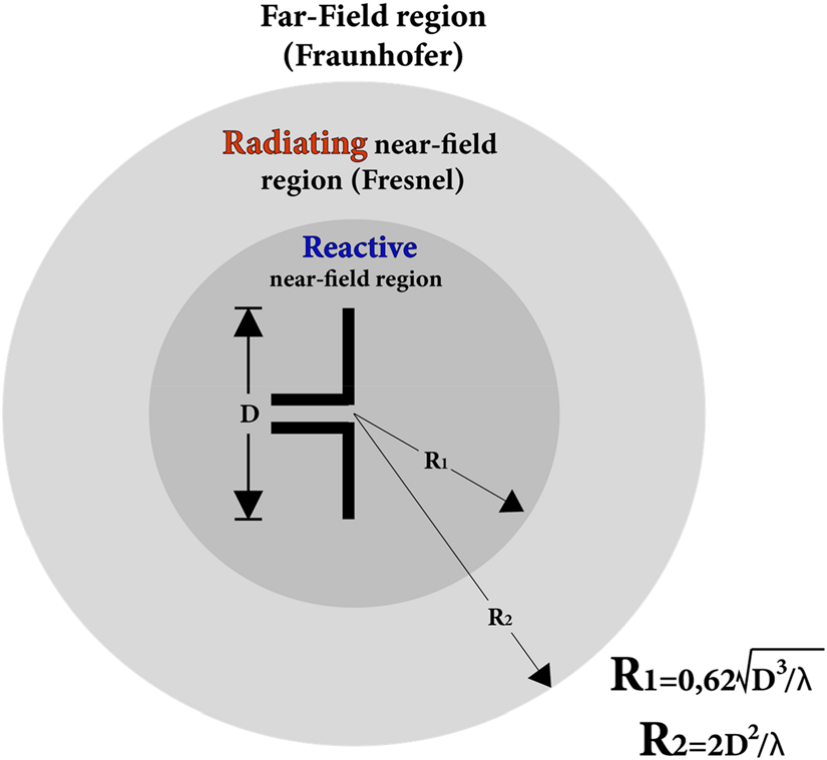
Near field and far field regions.

These field regions are illustrated in Fig. 17.

For this Yagi antenna, the dimension D is 63 mm and λ is 122.45 mm. Thus, the distant field region starts at approximately 65 mm, which indicates that the antennas should be separated by a distance greater than about 130 mm, at least. Nonetheless, due to a volume restriction of the equipment, the antennas were separated by less than 30 mm, which would make them operate in the near field region, which could lead to erroneous readings of the signal intensity.

Finally, we must have an antenna whose HPBW is the smallest possible, for this application, aiming to concentrate the RF signal as much as possible. It is worth mentioning that the total volume of the equipment limits this factor, as it does not allow us to have highly directive arrays, with many elements. The analyzed Yagi antenna has a 95° HPBW.

Thus, we conclude that the Yagi antenna was not suitable for use in *Osseus*, and another antenna, or an array of antennas, had to be proposed to meet all the criteria listed in this section.

### Project of the antenna and antenna array

From what was exposed in the previous section, we listed some parameters of the antenna in use to be improved. The antenna needs to be as small as possible to meet the portable device's final volume requirements. The antenna feed must be done in a more consistent way, via SMA connector soldered to it. The antenna needs an improvement in the gain, directivity, and, consequently, in the reduction of the HPBW. To guarantee certain radiation parameters, the antenna needs good impedance matching, along with a consistent way to input the signal of 2.45 GHz.

**Figure 18:**
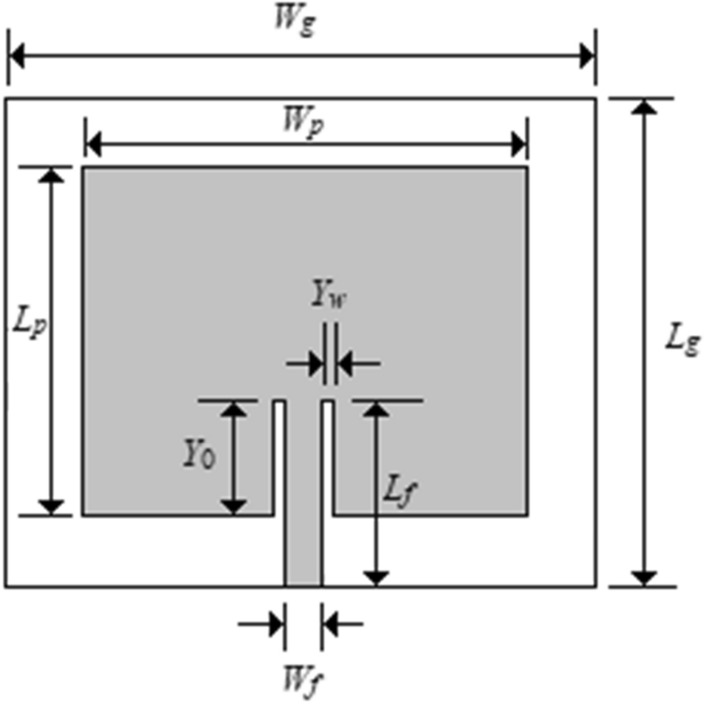
Microstrip patch antenna.

Based on the issues that need to be improved, a microstrip patch antenna and a patch antenna array have been proposed. As an initial proposal, we will start with a simple concept of a planar microstrip antenna with rectangular patch geometry. This geometry was chosen for its ease of design, since this application does not need such a sophisticated or robust antenna to function. Unlike the initial antenna, this antenna has a ground plane completely filled over the entire length of the dielectric. The chosen feed was via a microstrip line and the impedance matching with the input is made by inserting a line gap in the rectangular patch (Inset Fed). An illustration of this model can be seen in Fig. 18.

For the design of this type of antenna, the classic approach presented by Kumar and Shanmuganantham^15^ was used, which systematically describes how to design this kind of geometry. It is important to choose the appropriate dielectric material. In this approach, it is evident that the physical dimensions of the antenna can be reduced if we use a dielectric material with high relative electrical permittivity. Thus, the *ROGERS RO3010*^16^ was chosen, with ɛ_r_ = 10.2 and thickness h = 1.27 mm. The increase in ɛ_r_ tends to reduce the bandwidth, so we choose the largest thickness available from this manufacturer, compensating for this reduction^15^.

Initially, the dimensions of the rectangular patch^15^ are determined as:

2 $$W=\frac{c}{2{f}_{r}}\sqrt{\frac{2}{{\varepsilon }_{r}+1}}$$

3 $$L=\frac{c}{2{f}_{r}\sqrt{{\varepsilon }_{eff}}}+2\Delta L$$

In which,

4 $${\varepsilon }_{eff}= \frac{{\varepsilon }_{r}+1}{2}+\frac{{\varepsilon }_{r}-1}{2}{\left[1+12\frac{h}{W}\right]}^{-\frac{1}{2}} for \frac{W}{h}>1$$

5 $$\frac{\Delta L}{h}=\mathrm{0,412}\frac{\left({\varepsilon }_{eff}+\mathrm{0,3}\right)\left(\frac{W}{h}+\mathrm{0,264}\right)}{\left({\varepsilon }_{eff}-\mathrm{0,258}\right)\left(\frac{W}{h}+\mathrm{0,8}\right)}$$

Besides that, c is the speed of light in the vacuum, fr is the desired resonance frequency, *ε*_*eff*_ is the effective permittivity of the dielectric, and ∆L is the extended electrical length of the rectangular patch, due to the fringe effect^17^.

The next step is to design the gap in the patch, which will be responsible for the impedance matching for the antenna with its microstrip feed line. To do this, we need to calculate the patch impedance:

6 $${G}_{1}=\frac{1}{90}{\left(\frac{W}{{\lambda }_{0}}\right)}^{2} for W\ll {\lambda }_{0}$$

7 $${R}_{in}=\frac{1}{2{G}_{1}}$$

8 $${R}_{in}\left(y={y}_{0}\right)={R}_{in}\left(\frac{\pi }{L}{y}_{0}\right)$$

where G_1_ is the approximate conductance of one of the patch slots, R_in_ is the approximate resistance of the patch, R_in_ (y = y_0_) is the resistance of the patch after the insertion of the gaps and y_0_ is the length of the gap.

The width of the gap (Y_W_) can be approximated by a quarter of the width of the microfiche line (W_f_) that feeds the patch. According to the literature, this value usually brings a good impedance match. Therefore, by finding the approximate impedance of the patch and making the resulting impedance 50 Ω, for a correct match with the line of the same impedance, we project the inset fed.

Finally, we still need to design the microstrip line that will feed the patch. It should have a total length of an odd multiple of λ/4 and a width that results in an impedance of 50 Ω:

9 $${Z}_{linha}= \sqrt{{\varepsilon }_{eff}}\left[\frac{{W}_{0}}{h}+\mathrm{1,393}+\mathrm{0,667}lnln \left(\frac{{W}_{0}}{h}+\mathrm{1,444}\right)\right]$$

Thus, using the expressions above, the physical dimensions for the antenna are listed in Table 3.

**Table 3:** Dimensions of the antenna, microstrip line and *inset fed* (in mm).

Patch	Wp	25.8
	Lp	18.8
Entire dielectric	W_g_	30.0
	L_g_	30.0
Microstrip line	L_f_	16.8
	W_f_	2.0
Inset-fed	Y_w_	0.8
	Y_0_	8.0

**Figure 19:**
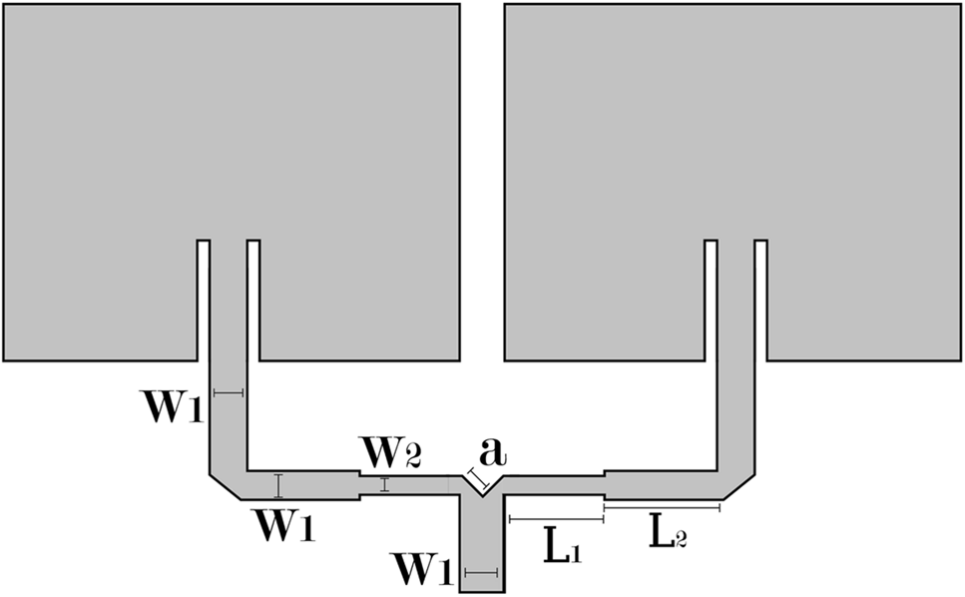
Antenna array and its proposed network feed.

The proposed array is illustrated in Fig. 19. In this case, the impedance matching will not be done by an inset fed, but by a network feed, whose physical dimensions are determined from the desired impedance values. The elements of the array are identical to the rectangular patch antenna.

The literature shows us that an array has as its basic characteristic an improvement in directivity and, consequently, the maximum gain along with the reduction of the HPBW, which are the parameters that we want to improve. Therefore, the design of the parallel array comes down to create a network feed that injects the signal in an equivalent way for two identical patches. This network feed must have a good impedance matching between each of its steps, to ensure that the injected signal reaches the radiator elements with the least possible loss^18,19^.

**Figure 20:**
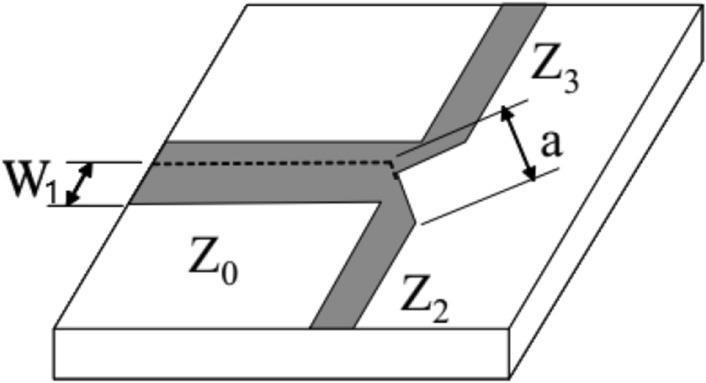
T-type junction.

Initially, it is necessary to design a power divider. There are many different models of power dividers, but the T-type junction was chosen for ease of design and manufacture. This junction can be seen in Fig. 20 and will serve to equally divide the power of the injected signal. In the T-junction, the impedance of each line determines its width, and the “V” shape with dimension “a” is necessary to minimize the parasitic reactances that arise due to the discontinuity of this junction.

In Visser^17^, it is recommended for “a” to be 1.8W_1_. Whenever there is a discontinuity the same rule will be applied. It is important to note that if we want to divide the signal intensity equally for both antennas, we need the junction lines to have twice the impedance of the line that receives the power. We can verify this through the following expressions:

10 $${P}_{1}={P}_{2}+{P}_{3}$$

11 $${P}_{2}=\frac{1}{1+{K}^{2}}{P}_{1}$$

12 $${P}_{3}=\frac{{K}^{2}}{1+{K}^{2}}{P}_{1}$$

13 $$\frac{{Z}_{2}}{{Z}_{3}}=\frac{{P}_{2}}{{P}_{3}}={K}^{2}$$

14 $$\frac{{Z}_{2}}{{Z}_{0}}=\frac{{P}_{1}}{{P}_{2}}=1+{K}^{2}$$

15 $${Z}_{2}=(1+{K}^{2}){Z}_{0}$$

16 $${Z}_{3}=(\frac{{1+K}^{2}}{{K}^{2}}){Z}_{0}$$

In which:

- *P*_1_ is the power delivered to the array;
- *P*_2_ is the power delivered to one of the elements of the array;
- *P*_3_ is the power delivered to the other element of the array;
- *K* is a factor that determines the proportion how the power will be divided (K=1 for equal division of power);
- *Z*_0_ is the characteristic impedance of the feed line;
- *Z*_2_ is the impedance of one of the sides of the T-junction;
- *Z*_3_ is the impedance of the other side of the T-junction.

**Figure 21:**
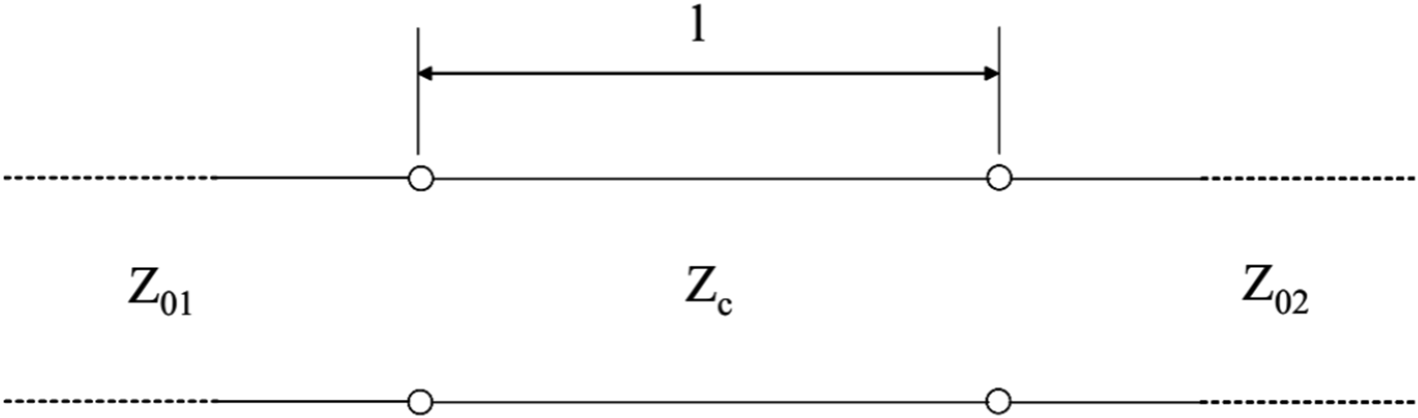
Insertion of λ/4 transformer.

As the lines that follow from the T-junction have an impedance different than 50 Ω, it is necessary to use an impedance transformer so that the network feed is matched with the antennas that we initially designed. The transformer used will be the one-quarter wavelength transformer. Its width is determined from the desired impedance, which is 50 Ω. Figure 21 illustrates the insertion of the λ/4 transformer.

To calculate the dimensions of the transformer, we consider the length λ/4 and the width obtained from (9), knowing that the impedance of the transformer must be:

17 $${Z}_{c}= \sqrt{{Z}_{01}{Z}_{02}}$$

in which *Z*_01_ is the impedance of the line of the T-junction and *Z*_02_ is the impedance of the antenna.

In this way, we completed the design of the array’s network feed by obtaining all of its physical dimensions. Lesser adjustments will need to be made, due to the fact that some of the expressions are approximate and, mainly, due to the fact that the high permissiveness we use to reduce the dimensions of the antenna causes a greater fringe effect.

Table 4 lists the dimensions of the network feed for this antenna array.

**Table 4:** Dimensions of the network feed of the array (in mm).

Dimension	Measure in mm
*L*_2_	12.2
*L*_3_	5.6
*W*_1_	2.0
*W*_2_	0.55
*W*_3_	2.0
*a*	1.0

## Data Availability

The datasets during and/or analyzed during the current study available from the corresponding author on reasonable request.

## Abbreviations

RF: Radio frequency
ISM: Industrial, scientific, and medical
SRR: Split-ring resonators
CPW: Coplanar waveguide
MTM: Metamaterial
HPBW: Half power beam width
SNR: Signal-to-noise ratio
DC: Direct current
mm: Millimeters
mV: MiliVolts
V: Volts
kHz: KiloHertz
MHz: MegaHertz
GHz: GigaHertz
dB: Decibels
dBi: Decibels isotropic
VSWR: Voltage standing wave ratio
Wifi: Wireless fidelity
SMA: Sub miniature version A
